# Realization of resistive switching and magnetoresistance in ZnO/ZnO-Co composite materials

**DOI:** 10.1038/srep31934

**Published:** 2016-09-02

**Authors:** Xiaoli Li, Juan Jia, Yanchun Li, Yuhao Bai, Jie Li, Yana Shi, Lanfang Wang, Xiaohong Xu

**Affiliations:** 1 Key Laboratory of Magnetic Molecules and Magnetic Information Materials of Ministry of Education and School of Chemistry and Materials Science, Shanxi Normal University, Linfen 041004, P. R. China; 2 College of Physics and Electronic Information, Shanxi Normal University, Linfen 041004, P. R. China

## Abstract

Combining resistive switching and magnetoresistance in a system exhibits great potential for application in multibit nonvolatile data storage. It is in significance and difficulty to seek a material with resistances that can be stably switched at different resistance states modulated by an electrical field and a magnetic field. In this paper, we propose a novel electrode/ZnO/ZnO-Co/electrode device in which the storage layer combines a nanostructured ZnO-Co layer and a ZnO layer. The device exhibits bipolar resistive switching characteristics, which can be explained by the accumulation of oxygen vacancies due to the migration of oxygen ions by external electrical stimuli and the contribution of Co particles in the ZnO-Co layer. Moreover, the magnetoresistance effect at room temperature can be observed in the device at high and low resistance states. Therefore, through electrical and magnetic control, four resistance states are achieved in this system, presenting a new possibility towards enhancing data densities by many folds.

Resistive random access memory (ReRAM) is considered as one of the most promising candidates for next-generation memories because of its simple structure, low cost, and high-density integration^1^,^2^,^3^. In general, a ReRAM device is usually composed of a storage layer sandwiched by two electrodes, and the device can be freely programmed into a high resistance state (HRS) or a low resistance state (LRS) under external electrical stimuli^3^. The transition from HRS to LRS is denoted as a setting process, and its opposite transition is denoted as a resetting process. The resistive switching (RS) behaviors of a large variety of functional materials including solid electrolytes, perovskites, binary oxides etc have been extensively explored. Among them, binary oxides such as TiO_2_, ZnO, and NiO etc. with simple compositions, low requirements for deposition techniques, and good compatibility with complementary metal-oxide semiconductor (CMOS) processes have attracted considerable attention^4^,^5^,^6^,^7^,^8^,^9^,^10^. At the same time, the discovery of giant magnetoresistance effect (GMR)^11^,^12^ has led to a great leap in the revolution of hard-drive data storage devices and the birth of spintronics. Thereafter, extensive studies on the magnetoresistance (MR) of heterogeneous structures including multilayers, magnetic tunnel junctions (MTJs), and granular films etc. have been performed with a view of potential applications in MR random access memory and magnetic sensors. Compared with layered films, granular films have some advantages, such as lower requirements for deposition techniques, ability to change the sizes of the magnetic nanoparticles and the microstructures of the films after deposition etc.^13^. Moreover, MR in granular films can work with a magnetic field applied in any direction. High MR ratios at room temperature (RT) have been found in nanostructured ZnO-Co granular films with current in the plane^14^,^15^,^16^,^17^.

Thus, both RS- and MR- based devices can be used in the field of random access memory. Recently, a new concept of combining RS and MR has received growing attention because of its potential applications in multibit nonvolatile data storage and artificial neuronal computing^18^,^19^,^20^,^21^. Realizing RS and MR in a material can further broaden the applications of random access memories encoding quaternary information. Therefore, seeking a material with resistances that can be stably switched at different resistance states modulated by electrical and magnetic fields is significant and difficult. From the abovementioned discussion, the RT MR effect has been achieved in nanostructured ZnO-Co films^15^,^16^,^17^, and highly reproducible RS properties have been achieved in ZnO systems^7^. This study proposes a novel Pt/ZnO/ZnO-Co/Au device, in which the storage layer combines a nanostructured ZnO-Co layer and a ZnO layer. The device can simultaneously show MR and RS effects at RT. Through electrical and magnetic control, four resistance states are realized in this system, presenting a new possibility to enhance data densities by many folds.

## Methods

**Figure 1:**
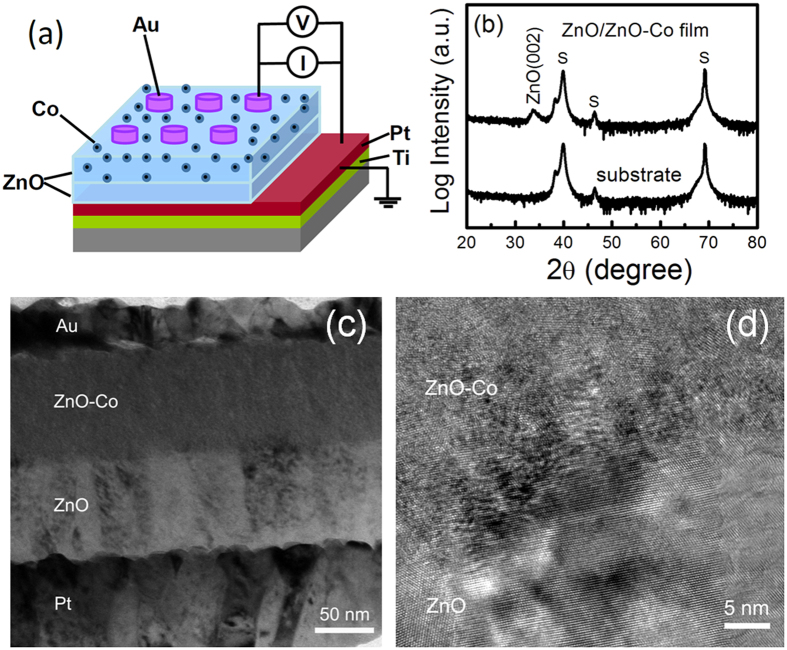
(**a**) A schematic structure of the Pt/ZnO/ZnO-Co/Au sample; (**b**) XRD patterns of the blank Si/SiO_2_/Ti/Pt substrate and the ZnO/ZnO-Co sample grown on it; (**c**) The cross-section TEM image of the Pt/ZnO/ZnO-Co/Au sample, where the Au and Pt layers are the top and bottom electrodes, respectively. (**d**) High resolution TEM image of the ZnO/ZnO-Co bilayer film.

A nanostructured ZnO-Co film with an underlayer of ZnO (approximately 80 nm) was deposited on the Si(100)/SiO_2_/Ti/Pt substrates by magnetron sputtering at RT. The nominal structure of the ZnO-Co film is [Co(0.6 nm)/ZnO(0.7 nm)]_50_; this structure was achieved by sequentially depositing an ultra-thin 0.6 nm Co layer and a 0.7 nm ZnO layer for 50 periods. Details of the growth have been described in a previous study^15^,^16^,^17^. Thereafter, the inert Au top electrode (TE) with diameter of 500 μm and thickness of about 40 nm was deposited with the aid of a shadow mask on the ZnO-Co film by magnetron sputtering. The inert Pt layer of the Si/SiO_2_/Ti/Pt substrate is used as the bottom electrode (BE). Figure 1(a) shows the schematic sample design of the Pt/ZnO/ZnO-Co/Au structure and the measurement setup. The current–voltage (*I*–*V*) characteristics were measured in a two-probe configuration by a Keithley 2400 semiconductor characterization system at RT, and during the measurements, the bias voltages were applied on the TE while the BE was grounded. In addition, MR at RT and the temperature dependence of the resistance were measured by a physical property measurement system (PPMS). The zero-field-cooled and field-cooled (ZFC-FC) magnetizations ranging from 2 K to 300 K in 100 Oe were measured with a superconducting quantum interference device magnetometer (SQUID). The structure of the sample was determined by X-ray diffraction (XRD) and transmission electron microscope (TEM).

## Results

Figure 1(b) compares the XRD patterns of the ZnO/ZnO-Co sample and the blank Si/SiO_2_/Ti/Pt substrate on the logarithmic scale. In addition to the peaks from the substrate, only a broad ZnO (002) peak is observed in the XRD pattern of the ZnO/ZnO-Co sample. No peaks related to Co may imply poor crystallinity or an amorphous state^15^,^16^,^17^,^22^, which can also be evidenced from the high-resolution TEM image shown in Fig. 1(d). The cross-sectional TEM image of the ZnO-Co-based sample shown in Fig. 1(c) clearly shows the layered structure of the sample, which is a ZnO/ZnO-Co bilayer film sandwiched by the Au and Pt electrode layers. Although the ZnO-Co layer was achieved by sequentially depositing a Co layer and a ZnO layer for 50 periods, the layered structure cannot be observed in the high-resolution TEM image of the ZnO-Co layer as shown in Fig. 1(d), which implies the formation of a ZnO-Co granular film instead of a multilayer. The result is consistent with our previous reports^15^,^16^,^17^. In addition, the clear ZnO lattice fringes can be observed in the ZnO layer, as shown in Fig. 1(d), whereas, the weak ZnO and Co lattice fringes observed in the ZnO-Co layer imply poor crystallinity or an amorphous state. For a ZnO-Co granular layer, the growth of ZnO and Co particles in the layer may be suppressed by each other. So the ZnO peak of the sample shown in Fig. 1(b) may be mainly from the ZnO layer of the sample. Therefore, the aforementioned TEM and XRD results illustrate the layered structure of the sample including an Au layer, a ZnO-Co granular layer, a ZnO underlayer on a layered substrate, and the poor crystallinity of the sample.

**Figure 2:**
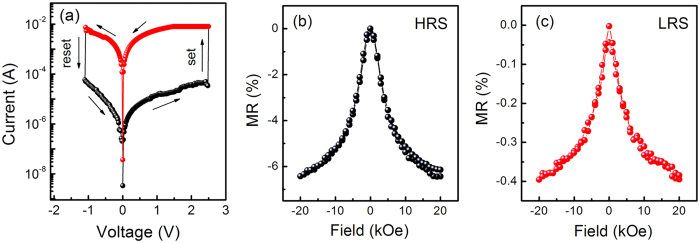
(**a**) The I–V curve of the ZnO/ZnO-Co sample in a semilogarithmic scale at RT. The arrows indicate the sweeping directions of the voltage. The field dependence of RT MR ratio of the sample at HRS (**b**) and LRS (**c**).

The key innovative results of our study are presented in Fig. 2(a–c), where the ZnO/ZnO-Co based sample shows bipolar RS characteristics, and the RT MR effect can be observed in the sample at HRS and LRS. Figure 2(a) displays the *I*–*V* characteristic of the sample at RT. A current compliance of 10 mA is applied to prevent dielectric breakdown of the sample. Figure 2(a) shows a positive bias voltage (*V*_set_) of approximately +2.48 V defined by the current flowing from the TE to the BE, which switches the cell from HRS to LRS (or *R*_ON_). In addition, a negative bias voltage (*V*_reset_) of −1.08 V is defined by the current flowing from the BE to the TE, which switches the cell from LRS to HRS (or *R*_OFF_). The memory window with the *R*_OFF_/*R*_ON_ ratio of 580 at a read voltage of 0.1 V can be discernible, enabling the periphery circuit to easily distinguish the storage information. Based on the aforementioned two resistance states of LRS and HRS achieved by electrical control, another two states for the sample can be further achieved by modulating a magnetic field. Figure 2(b,c) show the field dependence of the MR ratios of the ZnO-Co based sample at RT at HRS and LRS, respectively. The MR ratio is defined as [R(H)-R(0)]/R(0) × 100%, where R(H) and R(0) are the resistances in an external magnetic field and zero field, respectively. In our study, the maximum applied magnetic field is 20 kOe. The RT MR ratios of the ZnO-Co sample at HRS and LRS are −6.41%, and −0.40%, respectively. Although the MR ratio for the sample at LRS is dramatically reduced, it can still be maintained at RT. Thus, Fig. 2(b,c) imply that the four resistance states can be achieved in the nanostructured ZnO/ZnO-Co sample through the modulation of electrical and magnetic fields. In other words, the resistances of the ZnO/ZnO-Co sample can be divided into HRS and LRS by electrical stimuli because of the dependence of the past history of the electrical field on the resistance. Thereafter, the HRS and LRS can be involved in the two resistance states through the applied magnetic field. As this, four resistance states are achieved, namely, HRS-R(H), HRS-R(0), LRS-R(H), and LRS-R(0). In fact, the multiple resistance states have been previously observed in Co/CoO-ZnO/Co MTJs^20^,^21^, and MTJs with ferroelectric barriers^23^,^24^ etc. Comparatively, our ZnO/ZnO-Co composite sample has lower requirements for deposition techniques. Although the RT MR effect is relatively weak and needs to be enhanced in future work, it can provide a new clue to achieve multiple resistance states in a material through an easy and simple deposition method.

**Figure 3:**
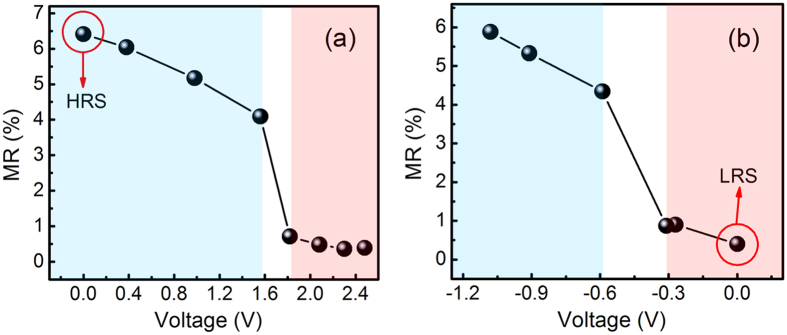
(**a**) The variation of the RT MR ratios of the ZnO/ZnO-Co sample at HRS with the applied positive voltages; (**b**) The variation of the RT MR ratios of the ZnO/ZnO-Co sample at LRS with the applied negative voltages.

The mentioned RT MR curves were measured after the sample was reset or set to HRS or LRS by applying *V*_reset_ of −1.08 V or *V*_set_ + 2.48 V, respectively. In further exploring the variation of MR in the sample during RS process, the RT MR curves were measured during the setting or resetting processes. For the sample at HRS, the MR curves were measured when several positive voltages lower than the *V*_set_ of 2.48 V were applied to the sample, namely, 0.38, 0.98, 1.56, 1.82, 2.08, and 2.30 V. Figure 3(a) shows the variation of MR ratios with the applied positive voltages. The figure shows that MR ratios are reduced when the applied positive voltages are increased. The opposite phenomenon was observed when several negative voltages lower than the *V*_reset_ of −1.08 V were applied to the sample at LRS, namely, −0.27, −0.31, −0.59, and −0.91 V. The MR ratios are enhanced with the increase of the applied negative voltage values as shown in Fig. 3(b). In fact, the process of continuously applying positive voltages on the sample at HRS is actually a setting process, and the process of applying negative voltages on the sample at LRS is a resetting one. According to the concept of the RS, the two processes lead to the decrease and increase of the resistances, which further lead to the reduction and enhancement of MR ratios. The phenomena imply that the sample with a larger resistance exhibits a higher MR ratio regardless of the application of positive or negative voltages. In addition, Fig. 3(a,b) show that the MR ratios are abruptly changed when the intermediate voltages are applied on the sample at HRS and LRS, whereas they vary slowly at the high and low voltages. Therefore, the high and low MR ratios for the sample during the RS process even possibly result from two different MR mechanisms. Thus, the subsequent discussion will focus on fitting the *I*–*V* curve and describing the dependence of the resistance of the ZnO/ZnO-Co sample at LRS and HRS on temperature to explain the RS and MR mechanisms and the relationship between them.

**Figure 4:**
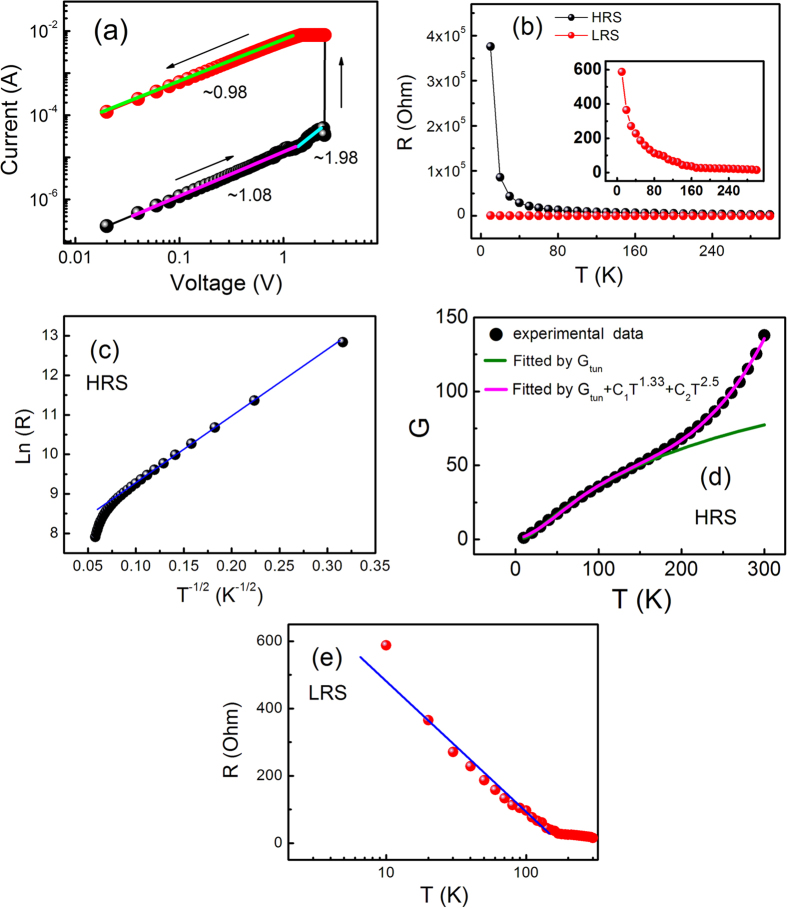
(**a**) The linear fitting for the I–V curve of the ZnO/ZnO-Co sample in double-logarithmic scale and the corresponding slope for each portion; (**b**) Temperature-dependence resistances of the sample at HRS and LRS, the inset shows an enlarged version for LRS; (**c**) The dependence of ln R on T^−1/2^ for the sample at HRS, the solid line represents the linear fit result; (**d**) Illustrations of the theoretical fits of conductivity as a function of temperature for the sample at HRS obtained from Equations 1 and 2; (**e**) The dependence of the resistance on logarithmic temperature for the sample at LRS, the solid line represents the linear fit result.

The positive voltage sweep region of the *I*–*V* curve of the ZnO/ZnO-Co sample shown in Fig. 2(a) is replotted in a double-logarithmic scale in Fig. 4(a). The fitting results are highlighted by colored lines at HRS and LRS. The figure shows that the *I*–*V* characteristics at HRS are complicated and can be divided into two regions. In the low voltage range of 0–1.23 V, the *I*–*V* relationship shows an almost linear dependence on the voltage with a slope of approximately 1.08. By contrast, the *I*–*V* characteristic exhibits a nonlinear behavior with a slope of approximately 1.98 when *V* > 1.23 V^25^,^26^. In contrast to the complicated scenario at HRS, the *I*–*V* curve presents a linear dependence on the voltage with a slope of approximately 0.98 at LRS, following the ohmic behavior and consistent with the formation of conductive filaments during the setting process^25^,^26^. Through the aforementioned fitting for the *I*–*V* curve, the RS process can be approximately divided into three parts. (1) Insulating regime. Although ZnO should be in an oxygen-deficient state in our ZnO/ZnO-Co sample, and it can be noted as ZnO_1−*x*_ because of the growth atmosphere of oxygen-deficiency, the sample is still insulating or semiconducting because of the observation of typical RS characteristics. The conduction at HRS may be mainly from the tunneling of electrons. This point can be evidenced from the temperature dependence of the resistance discussed later [Fig. 4(b–d)]. Under a positive voltage, oxygen ions may leave ZnO_1−*x*_ and cause it to have more oxygen vacancies (*V*_O_s). ZnO_1−*x*_ may be changed to ZnO_1−*x−y*_. The *y* values may be increased with the increase in the applied positive voltage. When the positive voltage is initially small, the *y* value and *V*_O_ concentration are small. Therefore, the wave functions of the electrons trapped in *V*_O_s are localized. And the double-logarithmic *I–V* relationship shows an almost linear dependence on the voltage in the range of low voltages in Fig. 4(a). (2) Intermediate regime. Through the continuous increase of the applied positive voltage, a considerable amount of *V*_O_s occurs, and parts of it may overlap with each other, delocalizing parts of the localized wave functions of the electrons trapped in *V*_O_s and resulting in the significant decrease of the resistance. This finding can be shown by the nonlinear *I*–*V* behavior with a slope of approximately 1.98 in the range of high voltages in Fig. 4(a). (3) Ohmic regime. When a large number of *V*_O_s trapping electrons overlap with one another and align along the direction perpendicular to the plane of the sample to form conductive filaments, the sample is completely switched to LRS. At this moment, the *I*–*V* curve presents a linear dependence on the voltage from 2.48 V to 0 V, as shown in Fig. 4(a). Related the *I–V* fitting results here with the variation of MR with the applied positive voltage described in Fig. 3(a), we can deduce that the high MR ratio may correspond to the insulating regime in which the MR effect arises from the tunneling mechanism. The low MR ratio may be consistent with the ohmic regime in which the weak MR effect may result from the GMR effect caused by the good conductivity.

In further analyzing the constitution of the filaments and the evidence in our proposal, the temperature dependence of the resistances for the sample at HRS and LRS [Fig. 4(b) and its inset] was measured. The resistances at HRS and LRS decrease with increasing temperature in the range of 10–300 K, implying semiconducting characteristics at the two states, which unambiguously demonstrate the key contribution of *V*_O_ to the conductive filaments^25^,^26^. In comparing the temperature dependence of the HRS resistance to that of the LRS resistance as shown in Fig. 4(b), the difference between them is also very obvious. The ln *R* vs T^−1/2^ plot for the sample at HRS shown in Fig. 4(c) indicates that ln *R* is almost linear to T^−1/2^, which is a typical characteristic of inter-particle spin-dependent tunneling in metal/insulator granular films^27^,^28^. We also convert the temperature dependence of resistance to the temperature dependence of conductivity (G), as shown in Fig. 4(d). The data were normalized to the conductivity at T = 10 K. In addition, they are fitted by the inter-particle tunneling conductivity described by Equation 1 and the conductivity combining the inter-particle tunneling and higher-order inelastic hopping described by Equation 2, respectively^16^,^29^,^30^,^31^.


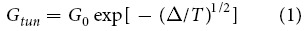


where *G*_*tun*_ is the tunneling conductivity, *G*_0_ is a free parameter, Δ = 4E/k_B_, E is the tunneling activation energy, and k_B_ is the Boltzmann constant.


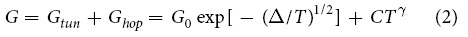


where *G*_0_ and *C* are free parameters, *γ* = *N* − [*N*/(*N* + 1)], *N* is the number of localized states in the barriers, and *G*_*hop*_ is the spin-independent higher-order inelastic hopping conductivity.

When T < 150 K, the conductivity of the sample as a function of temperature can be fitted well by Equation 1. When T > 150 K, the conductivity starts to deviate from Equation 1, which may imply that in addition to the tunneling conductivity, higher-order inelastic hopping caused by the presence of localized states within the ZnO matrix also contributes to the conductivity of the sample. Equation 2 fits our experimental data well with the second-order hopping (*γ* = 1.33) and the third-order hopping (*γ* = 2.5) at T > 150 K, as shown in Fig. 4(d). Therefore, the conduction of the film at HRS mainly has two channels: the spin-dependent tunneling channel, which results in a high RT MR effect, and the spin-independent high-order hopping, which is in agreement with our previous reports^16^.

Although the temperature coefficient of the resistance of the sample at LRS is negative below RT, showing a semiconductive-like transport behavior, the resistance increases gradually with decreasing *T* and shows a nearly logarithmic increase, as shown in Fig. 4(e). The reduction of the resistance at LRS and its gradual change with the measured temperature are expected to arise from the deficiency of oxygen ions and the accumulation of *V*_O_s in the system caused by the operation of the electric field. Thus, the deficiency of oxygen ions in the sample and its ohmic conductivity behavior result in the abrupt reduction of its MR because the Co particles are probably not separated completely by ZnO. Alternatively, the oxide interface between the Co particles does not provide enough insulation because of lack of oxygen. Similar phenomena have been observed in many systems. A small negative MR value was observed in a CoO-coated monodispersive Co cluster system when the oxygen gas-flow rate is low^13^,^32^. The oxygen-concentration dependence on the MR in a Co-Al-O granular system has also been investigated by Fujimori *et al.*^33^ The researchers observed a weak MR effect, and a small resistivity in the system with low oxygen concentration. Zhang *et al.*^21^ also contributed the weak MR in LRS in the Co/CoO-ZnO/Co magnetic tunnel junctions to the current-perpendicular-to-plane giant magnetoresistance effect caused by the migration of oxygen ions through the electrical manipulation.

**Figure 5:**
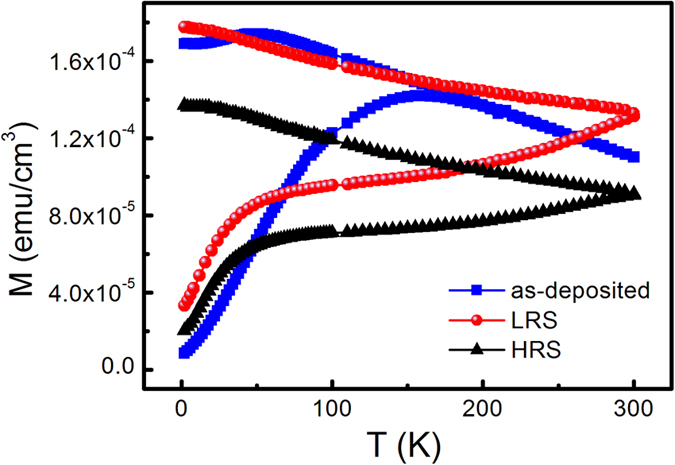
The ZFC-FC magnetizations of the ZnO/ZnO-Co sample at as-deposited state, LRS and HRS.

In addition to the key role of *V*_O_s, Co particles in the ZnO-Co layer of the sample may have a certain contribution to the formation of conductive filaments. The ZFC-FC magnetization is an effective way to analyze the existing states of Co in the film. Figure 5 compares the ZFC-FC magnetization curves of the sample at as-deposited state, LRS and HRS. The figure shows that at low temperatures, a large bifurcation is observed between the ZFC and FC magnetization curves, regardless of the three states, which are normally exhibited by superparamagnetic particles^16^,^17^,^34^. In addition, the blocking temperature (*T*_*b*_) of the as-deposited sample is approximately 160 K. Meanwhile, two *T*_*b*_ values appear for the sample at LRS: one is lower than 160 K, and the other is a high temperature of about 300 K. This finding is indicative of Co nanoparticles with two sizes in the sample at LRS. The only difference of the sample at LRS from the as-deposited one is the formation of conductive filaments driven by an electrical field. The Joule heating resulting from the electrical stimuli may cause the movements of Co atoms in the active regions. As a result, some Co particles in the active regions grow, and some Co particles in them diminish or disappear because of the movements of the Co between them. The size of Co particles located in the other regions except for the active ones may be unchanged. Thus, Co particles with at least two sizes may exist in the sample at LRS as shown in Fig. 5. Two similar *T*_*b*_ values still appear when the sample is reset from LRS to HRS, which may imply that Co particles cannot be reversibly changed during the resetting process. So it is reasonable to expect that during the resetting process, the conductive filament will dissolve from its thinnest and weakest part^3^. No appreciable effect is observed on the Co particle size during this process. Choi *et al.*^35^ also found the evolution of the size and shape of the Pt nanoparticles in the Pt dispersed SiO_2_ system because the electrically operated sample experienced sufficient Joule heating to cause Ostwald ripening. The broad peaks in the three ZFC magnetization curves shown in Fig. 5 imply a large size distribution of Co particles. In addition, the magnetizations of the sample at the as-deposited state, HRS and LRS are not exactly the same. Thus, the revolution of the size and shape of Co particles and the corresponding variation of the magnetization will be further investigated in the future.

**Figure 6:**
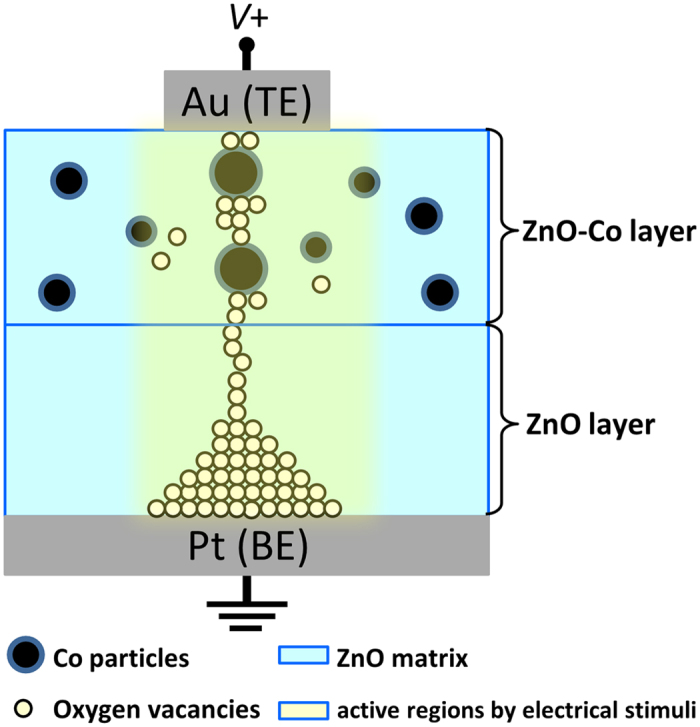
Schematic illustration of the conductive filament formed in the ZnO/ZnO-Co sample upon being subjected to a positive voltage.

In considering all of the aforementioned experimental results, the migration of the oxygen ions resulting in the accumulation of the *V*_O_s and the existence of Co in the ZnO-Co layer of the composite ZnO/ZnO-Co sample can be proposed to explain the observed RS behavior and the corresponding variation of the MR effect at HRS and LRS, as depicted schematically in Fig. 6. At HRS, the high MR ratio can be mainly attributed to the spin-dependent tunneling between the conducting magnetic Co particles through the ZnO semiconducting barriers in the ZnO-Co layers of the composite sample. Thereafter, because of the migration of the oxygen ions resulting in the accumulation of a high amount of *V*_O_s in the ZnO layer along the direction of the electric field, and because of the existence and evolution of the Co particles in the ZnO-Co layer caused by the electrical stimuli, the entire sample is switched from HRS to LRS under positive voltage, as shown in Fig. 6. Correspondingly, the Co particles in the ZnO-Co layer may be incompletely separated by the insulating ZnO because of the lack of oxygen ions. Moreover, even the size of some Co particles will change because of the Joule heating resulting from the electrical stimuli, which will lead to the abrupt reduction of its MR. With the growth mode from the BE to the TE, it is natural that the thinnest part of the formed conductive filament should be located near the TE^3^. When the TE is negatively biased, *V*_O_s in this region driven by the electric field rapidly migrate toward the TE. As a result, the concentration of *V*_O_s in the thinnest part of the conductive filament significantly decreases, leading to the rupture of the filament at that location and the formation of HRS^3^.

A sample with a composite ZnO/ZnO-Co storage layer was designed and grown by magnetron sputtering. It shows bipolar RS characteristics. The RT MR effect can be further achieved in the sample at HRS and LRS. Thus, multiple resistance states can be obtained in the sample by modulating the electrical and magnetic fields at RT. The RS may be caused by the accumulation of the *V*_O_s because of the migration of oxygen ions in the ZnO/ZnO-Co storage layer and the pre-existence of Co nanoparticles in the ZnO-Co layer. Our study can provide a new clue to achieve multiple resistance states in a material with an easy and simple deposition method. And the multiple resistance states may be achieved in other magnetic metal-insulator granular films.

## Additional Information

**How to cite this article**: Li, X. *et al.* Realization of resistive switching and magnetoresistance in ZnO/ZnO-Co composite materials. *Sci. Rep.* **6**, 31934; doi: 10.1038/srep31934 (2016).
